# Prevalence and correlates of antibodies to *Neospora caninum* in dogs in Portugal

**DOI:** 10.1051/parasite/2014031

**Published:** 2014-06-30

**Authors:** Carla Maia, Helder Cortes, Hugo Brancal, Ana Patrícia Lopes, Paulo Pimenta, Lenea Campino, Luís Cardoso

**Affiliations:** 1 Unidade de Parasitologia Médica, Instituto de Higiene e Medicina Tropical (IHMT), Universidade Nova de Lisboa (UNL) Lisboa Portugal; 2 Centro de Malária e outras Doenças Tropicais, IHMT-UNL Lisboa Portugal; 3 Faculdade de Medicina Veterinária, Universidade Lusófona de Humanidades e Tecnologias Lisboa Portugal; 4 Victor Caeiro Laboratory of Parasitology, Instituto de Ciências Agrárias e Ambientais Mediterrânicas, (ICAAM), University of Évora Portugal; 5 Clínica Veterinária da Covilhã Covilhã Portugal; 6 Faculdade de Ciências da Saúde, Universidade da Beira Interior Covilhã Portugal; 7 Escola Superior Agrária, Instituto Politécnico de Castelo Branco Castelo Branco Portugal; 8 Department of Veterinary Sciences, School of Agrarian and Veterinary Sciences, Universidade de Trás-os-Montes e Alto Douro (UTAD) Vila Real Portugal; 9 Centro de Ciência Animal e Veterinária (CECAV), UTAD Vila Real Portugal; 10 Hospital Veterinário de Trás-os-Montes Vila Real Portugal; 11 Departamento de Ciências Biomédicas e Medicina, Universidade do Algarve Faro Portugal

**Keywords:** *Neospora caninum*, Dog, cELISA, Prevalence, Portugal

## Abstract

Neosporosis, caused by *Neospora caninum*, is an important cause of abortion in cattle and of neurological disease in dogs. This study investigated the prevalence and correlates of antibodies to *N. caninum* in 441 dogs from the five regions of mainland Portugal. A commercial competitive enzyme-linked immunosorbent assay (cELISA) was used and specific antibodies were detected in 35 (7.9%) dogs. Seroprevalence levels were significantly different among some of the studied regions, as well as between stray dogs (13.6%) and hunting dogs (1.7%). The difference between seropositivity in dogs presenting musculoskeletal or neurological signs (21.4%) and that in animals without clinical signs compatible with neosporosis (5.6%) was close to statistical significance. This is the first report on the seroprevalence of *N. caninum* in dogs in Portugal. Neosporosis should be considered in the differential diagnosis of neurological disorders of dogs.

## Abstract (fr)

La néosporose, causée par *Neospora caninum*, est une cause importante d’avortement chez les bovins et de maladie neurologique chez les chiens. Cette étude porte sur la prévalence et les corrélations des anticorps contre *N. caninum* chez 441 chiens en provenance des cinq régions du Portugal continental. Un test commercial d’immuno-absorption enzymatique par compétition (cELISA) a été utilisé et des anticorps spécifiques ont été détectés dans 35 (7,9 %) des chiens. Les niveaux de séroprévalence étaient significativement différents entre certaines des régions étudiées, ainsi qu’entre les chiens errants (13,6 %) et les chiens de chasse (1,7 %). La différence entre la séropositivité des chiens présentant des signes musculo-squelettiques ou neurologiques (21,4 %) et des animaux sans signes cliniques compatibles avec la néosporose (5,6 %) était proche de la signification statistique. Cette étude est le premier rapport sur la séroprévalence de *N. caninum* chez les chiens au Portugal. La néosporose doit être considérée dans le diagnostic différentiel de désordres neurologiques des chiens.

## Introduction

*Neospora caninum* Dubey *et al*., 1988 [6] is a protozoan parasite of animals, with dogs (*Canis familiaris*) having an important role in the epidemiology of infection, as they and other related canids are the definitive hosts of this pathogen, and so shed oocysts into the environment [5, 7]. Neosporosis is an important cause of abortion in cattle, with high economic impact, and a neurological disorder in dogs [8].

Serological surveys indicate worldwide canine exposure to the parasite [5, 7]. *N. caninum* has been confirmed as a cause of bovine abortion in dairy herds in the North of Portugal [4, 12]. In serosurveys carried out in Holstein-Friesian cows in the Northern and Central regions of the country, seroprevalence was found to range from 28% in a random sample of dairy cattle to 46% in dairy herds with a history of abortion [3]. In Portugal, *N. caninum* has also been isolated from the faeces of a stray dog in the southern Algarve region [2].

The present study aimed at investigating the prevalence of antibodies to *N. caninum* in dogs countrywide, and to assess risk factors associated with infection or exposure in this host species, contributing to a better understanding of its epidemiology in Portugal.

## Material and methods

From January 2012 to December 2013, a total of 441 dogs from the five statistical regions of mainland Portugal were sampled in veterinary medical centres (388 domestic animals) and shelters (53 stray animals) and available data on correlates of infection were collected (Table 1). Dogs were randomly included after owners’ or legal holders’ informed consent. Stray animals had been housed to be sterilised for population control or to be given for adoption. This study was ethically approved by the ethical committee of the Universidade de Trás-os-Montes e Alto Douro as complying with the Portuguese legislation for the protection of animals (Law 92/1995).

**Table 1.** Prevalence of antibodies to *Neospora caninum* in dogs from Portugal as determined by a competitive ELISA.

Independent variable/category	No. of dogs tested (%)	Seroprevalence (%)	95% CI (%)
Region	441	*p* = 0.016	
North	88 (20.0)	10.2^a^	4.8–18.5
Centre	83 (18.8)	1.2^a^,^b^,^c^	0.03–6.5
Alentejo	110 (24.9)	5.5^d^	2.0–11.5
Lisbon	80 (18.1)	15.0^b^,^d^	8.0–24.7
Algarve	80 (18.1)	8.8^c^	3.6–17.2
Gender	430	*p* = 0.555	
Female	211 (49.1)	7.1	4.0–11.4
Male	219 (50.9)	9.1	5.7–13.7
Breed	426	*p* = 0.151	
Pure	256 (60.1)	6.3	3.6–9.9
Mongrel	170 (39.9)	10.6	6.4–16.2
Age (months)	409	*p* = 0.312	
[2–11]	32 (7.8)	12.5	3.5–29.0
[12–204]	377 (92.2)	7.7	5.2–10.9
Main aptitude	319	ND	
Pet	115 (36.1)	7.0	3.1–13.2
Hunting	59 (18.5)	1.7^e^	0.04–9.1
Stray	59 (18.5)	13.6^e^	6.0–25.0
Guard and watch	44 (13.8)	11.4	3.8–24.6
Farm and pastoral	21 (6.6)	4.8	0.6–16.2
Habitat	359	*p* = 0.745	
Urban	132 (36.8)	6.8	3.2–12.5
Rural	227 (63.2)	8.4	5.1–12.8
Housing	419	*p* = 0.137	
Totally indoors	36 (8.6)	0.0	0.0–9.7
In- and outdoors	157 (37.5)	10.2	5.9–16.0
Totally outdoors	226 (53.9)	8.4	5.1–12.8
Contact with other animals	405	*p* = 0.254	
No	98 (24.2)	5.1	1.7–11.5
Yes	307 (75.8)	9.4	6.4–13.3
Food	264	*p* = 0.257	
Strictly commercial	127 (48.1)	3.9	1.3–8.9
Including or strictly home-prepared	137 (51.9)	8.0	4.1–13.9
Compatible clinical signs	284	*p* = 0.050	
Absent	270 (95.1)	5.6	3.1–9.0
Present*	14 (4.9)	21.4	4.7–50.8
Total	441	7.9	5.4–10.6

a *p* = 0.018

b *p* = 0.003

c *p* = 0.032

d *p* = 0.049

e *p* = 0.032; ND: not determined/validated

* musculoskeletal or neurological.

Blood samples were obtained by cephalic or jugular venipuncture and serum was separated by centrifugation and preserved at −20 °C until used. A commercial competitive enzyme-linked immunosorbent assay (cELISA) was used for the detection of antibodies to *N. caninum* in serum samples according to the manufacturer’s instructions (VMRD, Pullman, WA, USA). Positive and negative control samples were provided in the kit. The percentage of inhibition (% I) was obtained by the formula: % I = 100 − [(sample OD × 100)/mean negative control OD]. When % was equal to or more than 30%, the sample was considered positive. Based on published data, the cELISA results correlated well with the indirect fluorescent antibody test [7, 8].

The exact binomial test was used to calculate confidence intervals (CI) for the proportions, with a 95% confidence level. The chi-square and Fisher’s exact tests compared proportions of seropositivity to *N. caninum* (no. of positive dogs/no. of dogs tested). A *p*-value < 0.05 was considered as statistically significant. Analyses were performed with SPSS^®^ 22 software for Windows.

## Results and discussion

Antibodies to *N. caninum* were detected in 35 (7.9%) out of the 441 dogs. Seroprevalence levels were significantly different among some of the studied regions (Table 1): the seroprevalence in dogs living in the Lisbon region was significantly higher than that in the Central and Alentejo regions, while seroprevalence in animals in the North and the Algarve was significantly higher than in those in the Centre. Seroprevalence in stray dogs was significantly higher than in hunting dogs (Table 1).

No statistical differences were found among the other independent variables/categories evaluated. However, the difference between seropositivity in dogs without clinical signs compatible with neosporosis (5.6%) and that in animals presenting musculoskeletal or neurological signs (21.4%) was very close to statistical significance (*p* = 0.05).

This is the first report on the seroprevalence of infection with or exposure to *N. caninum* in dogs in Portugal. A review of *N. caninum* infections in dogs worldwide revealed that among the risk factors, the lifestyle and age of the dogs were the most important. In fact, infection levels were higher in strays versus pets and in older versus young dogs [7, 8]. The higher prevalences documented in older dogs suggest most of them become infected after birth. In the present study, as well as in some others [10, 11, 13], no significant differences were observed between young and old animals. Nevertheless, it is important to point out that the number of dogs in the young group (i.e., 2–11 months old) was smaller than the adult group. Although no significant differences were observed in feeding habitats, the number of seropositive dogs was higher in those animals given food, including strictly home-prepared. Dogs are sometimes fed with raw or undercooked meat which might contain parasite cysts [9].

The geographical distribution of positive cases in the North of Portugal shown here is generally in accord with previous findings on bovine neosporosis [3], underlining the role of dogs in the life cycle of the parasite. On the other hand, the lowest prevalence obtained in dogs in the Centre of Portugal, a region where specific antibodies were previously reported in dairy cows [3], could be related to transplacentary transmission in cattle, with no direct participation of dogs in the epidemiological cycle [8]. Congenital transmission in dogs is clinically significant, but from an epidemiological perspective only a small proportion of dogs are congenitally infected [5].

Although the presence of specific antibodies in dogs only indicates that there was contact with the parasite, the presence of seropositive dogs in a particular area should be considered as a potential risk factor of *N. caninum* infection in cattle [8]. Thus, due to the lack of data, it is important to screen cattle from the regions of Alentejo, Algarve and Lisbon, where specific antibodies have been detected in dogs.

*Neospora caninum* can cause clinical disease in dogs of all ages [7]. In the present study an almost significant statistical association was found between seropositivity and musculoskeletal or neurological signs, suggesting that the disease should be considered in the differential diagnosis of neurological conditions in dogs [1].

## Conclusion

In conclusion, sanitary conditions and animal health must be improved to prevent the transmission risk of *N. caninum* by dogs. The results suggest including neosporosis in the differential diagnosis of neurological disorders of dogs, along with the need for further surveillance in relation to the influence of dogs on the epidemiology of infections with *N. caninum*.

## References

[1] Barber JS, Ham L, Polis I, Trees AJ. 1997 Seroprevalence of antibodies to *Neospora caninum* in Belgian dogs. Journal of Small Animal Practice, 38, 15–16 9121127 10.1111/j.1748-5827.1997.tb02978.x

[2] Basso W, Herrmann DC, Conraths FJ, Pantchev N, Vrhovec MG, Schares G. 2009 First isolation of *Neospora caninum* from the faeces of a dog from Portugal. Veterinary Parasitology, 159, 162–166 19036520 10.1016/j.vetpar.2008.10.025

[3] Canada N, Carvalheira J, Meireles CS, Costa JM, Rocha A. 2004 Prevalence of *Neospora caninum* infection in dairy cows and its consequences for reproductive management. Theriogenology, 62, 1229–1235 15325549 10.1016/j.theriogenology.2004.01.004

[4] Canada N, Meireles CS, Rocha A, Sousa S, Thompson G, Dubey JP, Romand S, Thulliez P, Costa JM. 2002 First Portuguese isolate of *Neospora caninum* from an aborted fetus from a dairy herd with endemic neosporosis. Veterinary Parasitology, 110, 11–15 12446085 10.1016/s0304-4017(02)00333-3

[5] Dubey JP. 2013 Neosporosis in dogs. CAB Reviews, 8(055), 1–26

[6] Dubey JP, Carpenter JL, Speer CA, Topper MJ, Uggla A. 1988 Newly recognized fatal protozoan disease of dogs. Journal of the American Veterinary Medical Association, 192, 1269–1285 3391851

[7] Dubey JP, Schares G. 2011 Neosporosis in animals – the last five years. Veterinary Parasitology, 180, 90–108 21704458 10.1016/j.vetpar.2011.05.031

[8] Dubey JP, Schares G, Ortega-Mora LM. 2007 Epidemiology and control of neosporosis and *Neospora caninum*. Clinical Microbiology Reviews, 20, 323–367 17428888 10.1128/CMR.00031-06 PMC1865591

[9] Gózdzik K, Wrzesien R, Wielgosz-Ostolska A, Bien J, Kozak-Ljunggren M, Cabaj W. 2011 Prevalence of antibodies against *Neospora caninum* in dogs from urban areas in Central Poland. Parasitology Research, 108, 991–996 21079995 10.1007/s00436-010-2143-0 PMC3058559

[10] Kuruca L, Spasojević-Kosić L, Simin S, Savović M, Lauš S, Lalošević V. 2013 *Neospora caninum* antibodies in dairy cows and domestic dogs from Vojvodina, Serbia. Parasite, 20, 40 24152767 10.1051/parasite/2013036 PMC3806349

[11] Rasmussen K, Jensen AL. 1996 Some epidemiologic features of canine neosporosis in Denmark. Veterinary Parasitology, 62, 315–317 10.1016/0304-4017(95)00867-5 8686180

[12] Thompson G, Canada N, Topa M, Silva E, Vaz F, Rocha A. 2001 First confirmed case of *Neospora caninum* associated abortion outbreak in Portugal. Reproduction in Domestic Animals, 36, 309–312 11928926 10.1046/j.1439-0531.2001.00307.x

[13] Trees AJ, Guj F, Tennant BJ, Balfour AH, Dubey JP. 1993 Prevalence of antibodies to *Neospora caninum* in a population of urban dogs in England. Veterinary Record, 132, 125–126 8447050 10.1136/vr.132.6.125

